# Association Between Nutritional Risk and Mental Health in Older Adults: Focusing on Depression and Cognitive Function

**DOI:** 10.3390/healthcare14081062

**Published:** 2026-04-16

**Authors:** Seohyeon Cho, Keon Woo, Yoonsoo Choy

**Affiliations:** 1 Department of Smart Healthcare Information, Healthcare Management, Eulji University, Seongnam-si 13135, Republic of Korea; 0707carrot@naver.com; 2 Department of Biohealth Industry, Graduate School of Transdisciplinary Health Sciences, Yonsei University, Seoul 03722, Republic of Korea; wwhh10@yuhs.ac

**Keywords:** older adults, nutritional risk, depression, cognitive function, frailty, mental health, DETERMINE checklist

## Abstract

**Highlights:**

**What are the main findings?**

- Nutritional risk was significantly associated with depression and cognitive function among older adults.
- Older adults with poorer nutritional status showed higher depressive symptoms and lower cognitive performance.

**What are the implications of the main findings?**

- Nutritional status should be considered an important factor in understanding mental health in older adults.
- Integrated management of nutrition may contribute to the promotion of mental health in the aging population.

**Abstract:**

**Background:** In the context of global population aging, nutritional risk has emerged as an important factor associated with both physical and mental health among older adults. This study aimed to examine the associations between nutritional risk, depression, and cognitive function in older adults and to explore potential variations across residential area, educational attainment, employment status, frailty status, and activities of daily living (ADL). **Methods:** Data were obtained from 9955 community-dwelling older adults aged 65 years and older who participated in the 2023 National Survey of Older Koreans. Nutritional risk was assessed using the DETERMINE checklist (21-point scale), a multidimensional screening tool reflecting dietary, functional, and social risk factors. Depression was measured using the Short-form Geriatric Depression Scale (15-point scale), and cognitive function was assessed using the Korean version of the Mini-Mental State Examination-2 (K-MMSE-2; 30-point scale). Hierarchical multiple linear regression, correlation, subgroup, and sensitivity analyses were conducted, adjusting for sociodemographic characteristics, health behaviors, and geriatric factors. **Results:** Correlation analyses showed significant associations between nutritional risk and cognitive function (r = −0.191, *p* < 0.05), nutritional risk and depression (r = 0.440, *p* < 0.05), and depression and cognitive function (r = −0.259, *p* < 0.05). Higher nutritional risk scores were significantly associated with greater depressive symptoms (B = 0.314, *p* < 0.001) and lower cognitive function (B = −0.051, *p* < 0.05). While some subgroup differences were observed, not all interaction effects reached statistical significance, and these findings should be interpreted with caution. **Conclusions:** These findings suggest that nutritional risk is associated with depressive symptoms and cognitive function in older adults. Given that the DETERMINE checklist reflects multidimensional vulnerability, the results should be interpreted as indicating broader risk contexts rather than direct nutritional status alone. These findings highlight the importance of integrated, multidimensional approaches to support older adults at nutritional risk in community settings.

## 1. Introduction

Global population aging is accompanied by a growing cumulative burden of chronic diseases, rapidly increasing care needs, and rising health and social welfare expenditures. An increasingly recognized driver of these trends is the double burden among older adults, in which undernutrition and overnutrition can coexist within the same population [1]. Nutritional problems are linked not only to a decline in physical function but also to poorer mental health, including cognitive decline and depression, leading to multidimensional health loss. As a result, nutrition in later life has become a key priority in both clinical practice and community health [2,3].

International evidence shows that a substantial proportion of community-dwelling older adults are malnourished or at risk, and that these groups often show a higher co-occurrence of cognitive impairment and depressive symptoms [2]. In a sample of free-living older adults in Cyprus, the prevalence of depression (18.6%) and cognitive decline (42.7%) was observed alongside the risk of undernutrition, which was significantly associated with lifestyle and environmental factors, such as diet, social activities, and body composition [3]. Evidence from Korea also supports an integrated approach: longitudinal community panel data have tracked cognitive decline over time, demonstrating that mental health factors, including depression and health behaviors, are key determinants of cognitive change—thereby strengthening the rationale for linking nutritional management with broader health promotion strategies [4]. Together, these findings suggest that nutrition management for older adults should be addressed systematically across community health and social welfare systems, rather than being limited to individual dietary choices [1].

In the 2022 Korea National Health and Nutrition Examination Survey, 18.2% of adults aged 65 years and older were reported to have inadequate nutrient intake. Similarly, the 2020 Survey of Older Koreans indicated that 19.0% of older adults required close attention to nutritional management and 8.8% required improvements in nutritional management [5]. Poor nutritional intake can lead to underweight status [6,7]. Weight loss, in particular, has been associated with higher risks of hip fracture and hospitalization, and has been reported to double mortality risk [8,9]. As nutritional risk can affect both physical and mental health in later life, monitoring and improving nutritional status among older adults remains a major public health priority.

In addition to physical health risks, mental health is a critical concern in older populations. Prior research has reported that approximately 80% of older adults with cognitive decline—considered an early stage of dementia—progress to dementia within six years [10]. As the proportion of older adults increases rapidly, dementia prevalence is also expected to rise [11]. Depression is another key mental health concern in later life. It is common among older adults, can be triggered or exacerbated by reduced physical activity, poor nutritional status, and social isolation, and can negatively affect perceptions of subjective health. Depression may further deteriorate overall health and progress to severe outcomes, including suicidality [12,13].

Cognitive decline and depression affect not only health but also everyday functioning. They can reduce the ability to perform instrumental activities of daily living (ADL/IADL), increasing the need for assistance and contributing to greater caregiving burden for patients and families, as well as higher social costs [14]. Beyond the difficulties in daily life, these conditions may also contribute to the worsening of physical health and a higher risk of nutritional risk.

When nutritional management is considered in relation to health in later life, its association with physical health is often examined first. Many previous studies have focused on physical outcomes, including evidence that nutritional status is associated with sarcopenia among older adults [15] and that depressive symptoms substantially worsen the effects of cognitive decline, slower gait, and deteriorating comorbidities on disability [16]. However, as the importance of mental health has gained greater attention, a growing body of research has examined the association between nutritional risk and mental health in older populations.

Previous research indicates that the risk of cognitive decline was approximately 1.4 times higher among older adults requiring nutritional attention or exhibiting poor nutritional status compared with those with good nutritional status [17]. Similarly, international evidence suggests a statistically significant difference in the prevalence of nutritional risk between individuals with and without depression [18]. Moreover, depressive symptoms and nutritional risk have been linked to other geriatric syndromes, including delirium, cognitive impairment, frailty, inadequate oral intake, and functional decline during hospitalization [19].

Although previous studies have documented associations between nutritional risk, depression, and cognitive function, findings have not always been consistent, particularly regarding the relative strength of these associations across different subpopulations. Many prior investigations have relied on regional or clinical samples or relatively small sample sizes, limiting generalizability. In addition, previous research has often focused on a single mental health outcome, without simultaneously considering multiple domains such as depression and cognitive function. Methodological variations in measurement and analytic approaches have further contributed to heterogeneity in reported findings, and heterogeneity across vulnerable subgroups has not been sufficiently examined.

To address these gaps, the present study uses a nationally representative sample of older adults in Korea to examine the associations between nutritional risk and both depression and cognitive function. In addition, subgroup analyses are conducted to explore heterogeneity across sociodemographic and geriatric characteristics, providing a more comprehensive understanding of vulnerable populations.

Using nationally representative data from the Survey of Older Koreans, this study aims to examine the associations between nutritional status and mental health outcomes, specifically depression and cognitive function, and to provide policy-relevant evidence. The specific objectives of this study are as follows:

1. To describe the status of nutrition management, depression, and cognitive function among older adults in Korea.
2. To identify sociodemographic and health behavior factors associated with depression and cognitive function.
3. To estimate the independent association of nutrition management with depression and cognitive function using multiple linear regression, adjusting for potential confounders.
4. To examine heterogeneity in these associations across subgroups defined by sociodemographic characteristics, older adult characteristics, and health behaviors, and to discuss policy implications for improving mental health through nutritional management among relatively vulnerable groups.

## 2. Materials and Methods

### 2.1. Data Source and Study Population

This study used data from the 2023 National Survey of Older Koreans, a nationally representative survey using a stratified, multistage cluster sampling design. Sampling weights were applied to obtain nationally representative estimates, as stratification and primary sampling unit identifiers were not available in the public-use dataset [20]. The National Survey of Older Koreans is a statutory survey conducted every three years since 2008, in accordance with Article 5 of the Welfare of Older Persons Act. This survey comprehensively examines the living conditions and needs of older adults, including health status, family and social relationships, economic and social activities, and residential environment. It provides foundational data for improving quality of life in later years, developing policies to address population aging, and supporting academic research.

The survey was conducted through face-to-face interviews using the Tablet-PC Assisted Personal Interview (TAPI) method by 176 trained interviewers, based on a questionnaire designed by the research team. The survey period was from 4 September to 12 November 2023. Based on the designed sampling method, the survey was completed by 10,178 adults aged 65 years and older from 7605 households across 977 survey districts. After excluding incomplete data, data from 10,078 individuals (including 123 proxy respondents) from 7556 households were included in the analysis. Of these, the 123 proxy respondents were subsequently excluded, resulting in a final analytic sample of 9955 older adults who responded directly to the survey.

### 2.2. Study Variables

#### 2.2.1. Dependent Variables: Mental Health in Older Adults

The dependent variables were (1) depression and (2) cognitive function. Depression was measured using 15 items with binary (yes/no) responses, including questions such as “Are you basically satisfied with your life?”, “Have you dropped many of your activities and interests?”, and “Do you feel that your life is empty?” A score of 1 was assigned to responses of “yes” and 0 to “no.” Items 1, 5, 7, 11, and 13, for which a “yes” response indicated the absence of depression, were reverse-coded. The total score ranged from 0 to 15, with higher scores indicating greater depressive symptoms (Supplementary S2).

Depression was assessed using the 15-item Geriatric Depression Scale (GDS-15), a widely used screening tool for depressive symptoms in older adults [21]. The Korean version of the GDS-15 has been validated and shown to have acceptable reliability and validity in older populations [22]. Cognitive function was assessed using the total score of the Korean version of the Mini-Mental State Examination-2 (K-MMSE-2), administered through a separate assessment form. It was evaluated across multiple domains, including registration and recall, orientation to time and place, attention and calculation, and language. Each item was scored as either incorrect (0) or correct (1). The total score ranged from 0 to 30, with higher scores indicating better cognitive function.

#### 2.2.2. Independent Variable: Nutritional Risk in Older Adults

The independent variable, nutritional risk, was measured using a scale based on the DETERMINE (Determine Your Nutritional Health) checklist developed by the Nutrition Screening Initiative (NSI) [23]. The checklist covers nine domains: disease, eating poorly, tooth loss/mouth pain, economic hardship, reduced social contact, multiple medicines, involuntary weight loss/gain, need for assistance in self-care, and age above 80 years. This checklist was used as the assessment criterion in the “Nutritional Management” section of the 2023 National Survey of Older Koreans (Supplementary S1).

The DETERMINE checklist is a screening tool designed to identify nutritional risk in older adults and comprises multiple items reflecting diverse risk factors, including dietary intake, health conditions, and functional limitations, rather than representing a single underlying construct of nutrition. Given its purpose as a risk screening instrument, the total score was used as a continuous independent variable in the main analyses to capture the cumulative burden of nutritional risk. In addition, item-level analyses were conducted to explore the specific nutritional risk factors most strongly associated with mental health outcomes.

While some DETERMINE items may conceptually relate to age and functional status variables, the checklist primarily captures nutritional risk, whereas age, ADL, and frailty represent broader demographic and functional characteristics. These constructs are associated but not identical and therefore reflect distinct theoretical domains.

Some items in the DETERMINE checklist may conceptually overlap with covariates such as age, functional status (ADL), and frailty. However, the DETERMINE checklist is designed to assess overall nutritional risk, whereas these covariates represent broader demographic and health-related characteristics. Although related, these constructs are not identical and were therefore included simultaneously in the models to account for potential confounding effects.

The original instrument consisted of 12 items, including “I have an illness or condition that made me change the kind and/or amount of food I eat,” “I eat fewer than two meals per day,” and “I have tooth or mouth problems that make it hard for me to eat.” Item 3, which addressed food variety, was originally divided into three separate questions by food type; these were consolidated into a single item: “I eat few fruits, vegetables, or milk products.” The remaining items were then combined, yielding a final 10-item scale. Weights were assigned to each item according to the original scoring criteria, resulting in a total possible score of 21 points (Supplementary Table S4). For the nutritional risk scale, a score of 0 was assigned to “no” responses, while weighted scores were assigned to “yes” responses based on the original scoring system. Higher scores indicated poorer nutritional status.

Although the DETERMINE checklist was originally developed as a screening tool rather than a diagnostic instrument, it has been applied in Korean national survey settings to assess nutritional risk among community-dwelling older adults. Internal consistency of the DETERMINE checklist was assessed using Cronbach’s alpha (Supplementary Table S2). Given that the checklist comprises diverse nutritional risk indicators rather than items representing a single underlying construct, relatively modest internal consistency is expected and should be interpreted with caution.

#### 2.2.3. Control Variables

To identify factors associated with mental health in older adults, control variables were selected based on variables and categories used in previous research. These included sex, age, residential area, educational attainment, smoking status in the past year, alcohol consumption status, perceived health status, number of chronic diseases, current employment status, living arrangements, frailty status (FRAIL scale), regular exercise, disability status, number of healthcare facility visits, and activities of daily living (ADL).

Age was categorized into four groups: 65–69, 70–79, 80–89, and 90 years and older. Residential area was classified as urban or rural. Educational attainment was categorized as primary education (elementary school or below), secondary education (middle or high school), and tertiary education (college or above). Smoking status and alcohol consumption in the past year were each measured as binary variables (smoker/nonsmoker; drinker/nondrinker).

Perceived health status was measured using a 5-point Likert scale (very good, good, moderate, poor, and very poor), with higher scores indicating better self-perceived health. The number of chronic diseases was categorized as 0, 1, 2, 3, or more. Current employment status was a binary variable (currently working/not working). Living arrangements were classified as living alone or living with others.

Frailty status was categorized as robust, pre-frail, or frail according to criteria established in previous research. Regular exercise and disability status were each measured as binary variables (yes/no). The number of healthcare facility visits in the past month was dichotomized as fewer than two visits or two or more visits, based on the sample mean. This categorization was applied to simplify the analysis and enhance interpretability.

ADL (Activities of Daily Living) was assessed using seven items: “dressing,” “washing face, brushing teeth, and shampooing,” “bathing or showering,” and others. Each item was scored as follows: 2 points for complete independence, 1 point for partial assistance, and 0 points for complete assistance, yielding a total scale score of 14 points. Respondents who selected “complete independence” for all items (total score of 14) were classified as “completely independent,” while those with scores below 14 were classified as “dependent.” Activities of daily living (ADL) were dichotomized into independent and dependent groups based on functional status to facilitate interpretation.

### 2.3. Statistical Analysis

Data analysis was performed using IBM SPSS Statistics version 23.0. The distribution of participants’ individual characteristics and main variables was summarized using frequency counts and percentages. The status of nutritional risk, depression, and cognitive function scores was described using means and standard deviations.

All categorical variables were entered as dummy variables in the regression models. Certain variables were dichotomized to align with the original survey categorization and to enhance interpretability in regression analyses; however, we acknowledge that dichotomization may reduce variability and statistical power.

All analyses were conducted using the Complex Samples module in SPSS, incorporating sampling weights to obtain nationally representative estimates. As stratification and primary sampling unit (PSU) identifiers were not available in the public-use dataset, full adjustment for complex design effects was not possible. This limitation is acknowledged in the interpretation of statistical inference.

Hierarchical multiple linear regression analyses were conducted to examine the associations between nutritional risk and the outcomes of depression and cognitive function, while adjusting for covariates. The analyses were conducted in three steps. Model 1 included sociodemographic variables, Model 2 additionally included health-related variables, and Model 3 further included nutritional risk (DETERMINE score). This hierarchical approach allowed us to examine the incremental contribution of nutritional risk beyond sociodemographic and health-related factors. Interaction terms between nutritional risk and selected subgroup variables (residential area, educational attainment, employment status, frailty status, physical activity, and ADL) were included to assess potential effect modification. In addition, sensitivity analyses were conducted to assess the robustness of the findings.

Given the number of subgroup and item-level analyses performed, findings were interpreted cautiously considering the potential for type I error.

To examine differences in depression and cognitive function according to participants’ individual characteristics, independent *t*-tests and one-way analysis of variance (ANOVA) were conducted, followed by Scheffé’s post hoc test to identify significant differences between group means. Additionally, differences in depression and cognitive function according to the 10 nutritional management items were analyzed using independent *t*-tests to identify which items were most strongly associated with mental health outcomes in older adults.

Given that the outcome variables, depression and cognitive function, were measured as continuous scores, linear regression models were considered appropriate for examining their associations with nutritional risk. The assumptions of linear regression were evaluated prior to analysis, and the models were deemed suitable for the data.

Pearson correlation coefficients were calculated to examine the relationships among continuous variables, including nutritional risk, depression, and cognitive function. Hierarchical multiple linear regression analyses were then performed to examine the associations between nutritional risk and depression and cognitive function after adjusting for individual characteristics.

Internal consistency was assessed using Cronbach’s alpha for both the DETERMINE checklist and the GDS-15 (Supplementary Tables S2 and S3). It should be noted that the DETERMINE checklist comprises diverse nutritional risk indicators rather than items representing a single underlying construct; therefore, relatively modest internal consistency is expected and should be interpreted with caution.

Subgroup analyses were additionally conducted to examine whether the association of nutritional risk with depression and cognitive function differed according to residential area, educational attainment, current employment status, frailty status, regular exercise, and ADL. Unstandardized regression coefficients (B) are reported for subgroup analyses. Interaction terms between nutritional risk and each potential moderator were entered into the fully adjusted regression models to assess effect modification. Diagnostic checks indicated no major violations of linear regression assumptions.

Participants with missing data (*N* = 123) were excluded from the analysis, and a complete-case approach was applied. No extreme outliers were identified that required exclusion from the analysis.

Sampling weights were applied to account for the complex survey design. However, as stratification and primary sampling unit (PSU) identifiers were not available in the dataset, a fully specified complex sample design could not be implemented. Therefore, standard errors may be underestimated, and the results should be interpreted with caution.

### 2.4. Ethical Considerations

This was a secondary data analysis using data from the 2023 National Survey of Older Koreans. The data were anonymized by the Korea Institute for Health and Social Affairs, with no personally identifiable information. This study was approved by the Institutional Review Board (EUIRB2025-332).

## 3. Results

### 3.1. General Characteristics of Study Participants

The general characteristics of the participants are presented in Table 1. Among the 9955 older adults, 44% were male and 56% were female. Regarding age distribution, 34.8% were aged 65–69 years, 41.1% were aged 70–79 years, 21.7% were aged 80–89 years, and 2.4% were aged 90 years and older. Regarding residential areas, 73.9% resided in urban areas and 26.1% resided in rural areas. Educational attainment was distributed as follows: primary education, 40.1%; secondary education, 52.8%; and tertiary education, 7.0%.

Regarding health behaviors, 9.4% were smokers and 90.6% were non-smokers, whereas 37.6% consumed alcohol and 62.4% did not. For perceived health status, 2.7% reported being “very good,” 40.0% “good,” and 34.2% “moderate.” Regarding chronic diseases, 14.1% had no chronic diseases, and 22.2% had one chronic disease. Regarding current employment status, 39.4% were currently working and 60.6% were not working.

Concerning living arrangements, 33.0% lived alone, and 67.0% lived with others. Regarding frailty status, 61.6% were classified as robust, 31.4% as pre-frail, and 7.0% as frail, indicating that approximately 40% of older adults experienced some degree of frailty progression. More than half (53.0%) of the participants reported engaging in regular exercise. Only 3.8% had received a disability determination. Regarding healthcare facility visits in the past month, 66.1% reported fewer than two visits, whereas 33.9% reported two or more visits. With respect to ADL dependency, 92.1% were completely independent and 7.9% were dependent.

The mean nutritional risk score was 2.27 out of 21 points, the mean cognitive function score was 24.61 out of 30 points, and the mean depression score was 3.12 out of 15 points.

### 3.2. Differences in Mental Health According to General Characteristics and Nutritional Management Items

The results of the analysis of the differences in depression and cognitive function according to general characteristics and nutritional management items are presented in Supplementary Table S1.

#### 3.2.1. Differences According to General Characteristics

With respect to age, those aged 90 years and older had depression scores of 5.34 (SD = 3.81) and cognitive function scores of 19.32 (SD = 5.61), while those aged 80–89 years had scores of 4.07 (SD = 3.51) and 22.04 (SD = 5.04), respectively. Those aged 65–69 years had the lowest depression scores of 2.40 (SD = 2.82) and the highest cognitive function scores of 26.28 (SD = 4.31). Post hoc analysis revealed that depression was highest among those aged 90 years and older, followed by those in their 80s, 70s, and 60s in descending order. Cognitive function showed the opposite pattern, being highest in the 60s age group and lowest in the 90s age group.

Educational attainment exhibited a similar pattern. Those with primary education had depression scores of 3.81 (SD = 3.51) and cognitive function scores of 22.52 (SD = 4.80), while those with tertiary education had scores of 2.09 (SD = 2.80) and 27.50 (SD = 2.36), respectively. Post hoc analysis indicated that depression was highest among those with primary education and lowest among those with tertiary education, whereas cognitive function showed the opposite pattern.

Regarding perceived health status, those reporting “very good” health had depression scores of 1.48 (SD = 2.05) and cognitive function scores of 26.87 (SD = 2.61), those reporting “good” health had scores of 1.76 (SD = 2.24) and 26.01 (SD = 4.12), and those reporting “very poor” health had scores of 7.78 (SD = 3.99) and 19.70 (SD = 5.81), respectively. Post hoc analysis revealed that depression was highest among those reporting “very poor” health, with no significant difference between the “very good” and “good” groups. For cognitive function, scores were highest among those reporting “very good” health, followed by “good,” with “very poor” showing the lowest scores.

With respect to chronic diseases, those with no chronic diseases had depression scores of 1.67 (SD = 2.42) and cognitive function scores of 26.18 (SD = 4.08), while those with 3 or more chronic diseases had scores of 4.37 (SD = 3.56) and 23.61 (SD = 4.79), respectively. Post hoc analysis indicated that depression was highest among those with three or more chronic diseases, while cognitive function was highest among those with no chronic diseases.

Regarding frailty status, robust older adults had depression scores of 2.21 (SD = 2.55) and cognitive function scores of 25.66 (SD = 4.53), pre-frail older adults had scores of 3.95 (SD = 3.31) and 23.40 (SD = 4.78), and frail older adults had scores of 7.42 (SD = 3.85) and 20.86 (SD = 5.41), respectively. Post hoc analysis revealed that depression was highest among frail older adults, followed by the pre-frail and robust groups. Cognitive function showed the reverse order: robust, pre-frail, and frail.

#### 3.2.2. Differences According to Nutritional Management Items

Analysis of differences in nutritional management items revealed that depression scores were generally higher among those who responded “yes” to each item. However, for the item “drinking 3 or more glasses of alcohol almost every day,” the “no” response group showed higher depression scores. Similarly, for cognitive function, “yes” responses were associated with lower scores for most items, but the “drinking 3 or more glasses of alcohol almost every day” item showed significantly higher cognitive function scores in the “no” response group. Although a few items, such as “changing the amount or type of food” were not statistically significant for cognitive function, most items showed significant results.

### 3.3. Correlation Analysis

The results of the correlation analyses are presented in Table 2. Depression and cognitive function showed a statistically significant negative correlation (r = −0.259, *p* < 0.05). Nutritional risk and depression showed a statistically significant positive correlation (r = 0.440, *p* < 0.05). A statistically significant negative correlation was also found be- tween Nutritional risk and cognitive function (r = −0.191, *p* < 0.05).

### 3.4. Associations Between Nutritional Risk and Mental Health in Older Adults

The results of the hierarchical multiple regression analyses examining the association between nutritional risk and mental health, after adjusting for sociodemographic characteristics, are presented in Table 3 and Table 4. Multicollinearity diagnostics indicated that variance inflation factor (VIF) values did not exceed 10, suggesting no significant multicollinearity among the variables.

#### 3.4.1. Association of Nutritional Risk with Depression and Cognitive Function

The analysis showed that each one-point increase in the nutritional risk score was associated with a 0.314-point increase in depression (*p* < 0.001; 95% CI: 0.313, 0.315) and a 0.051-point decrease in cognitive function (*p* < 0.001; 95% CI: −0.052, −0.050), after adjusting for covariates in the hierarchical regression model.

#### 3.4.2. Sociodemographic Characteristics and Mental Health

In Model 3, where nutritional risk was additionally included (Model 3), females had lower depression (B = −0.143, *p* < 0.001) and lower cognitive function (B = −0.575, *p* < 0.001) compared to males. With respect to age, depression was lower among individuals in their 70s and 80s compared to those in their 60s, whereas those aged 90 years and older showed higher depression, and this difference was statistically significant (*p* < 0.001). Cognitive function significantly decreased with increasing age (*p* < 0.001).

Regarding residential area, older adults living in rural areas had lower depression (B = −0.376, *p* < 0.001) and lower cognitive function (B = −1.543, *p* < 0.001) compared to those living in urban areas. In terms of educational attainment, individuals with primary education had higher depression (B = 0.054, *p* < 0.001) and lower cognitive function (B = −2.568, *p* < 0.001) compared to those with Higher education. Those with secondary education also showed significantly lower cognitive function (*p* < 0.001), and depression was also significantly associated with educational level.

In Model 3, where nutritional risk was additionally included (Model 3), smokers had higher depression (B = 0.063, *p* < 0.001) and lower cognitive function (B = −0.263, *p* < 0.001) compared to non-smokers. Compared to non-drinkers, drinkers showed lower levels of depression (B = −0.072, *p* < 0.001) and cognitive function (B = −0.210, *p* < 0.001).

Better perceived health status was associated with lower depression (B = −0.990, *p* < 0.001) and higher cognitive function (B = 0.571, *p* < 0.001).

Regarding chronic diseases, depression was significantly associated with the number of chronic conditions (*p* < 0.001). For cognitive function, individuals with two chronic conditions exhibited the greatest decline, whereas those with three or more conditions showed a relatively smaller decrease.

Regarding employment status, non-working older adults had higher depression (B = 0.588, *p* < 0.001) and lower cognitive function (B = −0.506, *p* < 0.001) compared to working older adults. For living arrangements, those living alone had higher depression (B = 0.130, *p* < 0.001) and lower cognitive function (B = −0.375, *p* < 0.001) compared to those living with others.

The number of healthcare facility visits in the past month was significantly associated with both depression and cognitive function (*p* < 0.001). In addition, older adults who did not exercise regularly had higher levels of depression and lower cognitive function than those who exercised regularly (*p* < 0.001).

Those with disabilities had higher depression (*p* < 0.001), whereas the association with cognitive function was not statistically significant (*p* > 0.05). Compared to robust older adults, those who were prefrail and frail showed higher levels of depression and lower cognitive function (*p* < 0.001). Finally, for ADL, dependent older adults had higher depression (B = 0.705, *p* < 0.001) and lower cognitive function (B = −1.573, *p* < 0.001) than completely independent older adults.

### 3.5. Association of Individual Nutritional Management Items with Mental Health

The association of individual nutritional management items with depression and cognitive function in older adults is presented in Table 5.

### 3.6. Subgroup Analysis: Nutritional Risk and Mental Health Across Sociodemographic and Geriatric Characteristics

#### 3.6.1. By Residential Area, Educational Attainment, and Employment Status

Subgroup analyses were conducted to examine whether the association between nutritional risk on depression and cognitive function differed according to residential area, educational attainment, and employment status (Table 6).

For residence, the interaction term was not statistically significant for depression (B = −0.017, SE = 0.029, *p* = 0.571), but was statistically significant for cognitive function (B = 0.234, SE = 0.060, *p* < 0.001).

Regarding educational attainment, no statistically significant interaction was observed for depression. For cognitive function, the interaction term was significant only for the secondary education group (B = 0.123, *p* = 0.036), whereas the primary education group was not statistically significant.

For employment status, the interaction term was statistically significant for depression (B = 0.078, SE = 0.030, *p* = 0.010) and for cognitive function (B = −0.106, SE = 0.044, *p* = 0.015).

#### 3.6.2. By Frailty Status, Exercise Habits, and ADL

Subgroup analyses were conducted to examine whether the association of nutritional risk with depression and cognitive function differed according to frailty status, regular exercise habits, and ADL.

Subgroup analyses were also conducted according to frailty status, regular exercise habits, and ADL. For frailty status, none of the interaction terms were statistically significant for either depression or cognitive function.

Regarding physical activity, no statistically significant interaction associations were observed for depression or cognitive function. Similarly, for ADL, the interaction terms were not statistically significant for either depression or cognitive function.

Given the number of subgroup and item-level analyses conducted, these findings should be interpreted with caution due to the potential risk of type I error.

## 4. Discussion

This study examined the association between nutritional risk and mental health outcomes, including depression and cognitive function, among older adults using hierarchical multiple regression analyses. In the fully adjusted model, higher levels of nutritional risk were significantly associated with increased depressive symptoms and decreased cognitive function. Nutritional risk was significantly associated with both depression and cognitive function, with a stronger association observed for depression. The association with cognitive function was statistically significant but relatively small in magnitude. Subgroup patterns were observed in certain groups; however, these findings should be interpreted with caution, as not all interaction associations reached statistical significance. These findings remained consistent after controlling for a wide range of sociodemographic and health-related variables, supporting the robustness of the observed associations. The hierarchical regression analysis further demonstrated that Nutritional risk contributed additional explanatory power beyond sociodemographic and health-related factors, supporting its independent association with mental health outcomes.

The findings indicate that the association with cognitive function was weaker compared to that observed for depression. These results suggest that nutritional status may play an important role in the emotional well-being of older adults. In addition, subgroup group differences were observed in specific subgroups, although not all interaction associations reached statistical significance.

Nutritional risk was positively associated with depressive symptoms, and higher levels of nutritional risk were linked to increased depression scores. These findings are consistent with previous studies conducted among older adults in Cyprus [3] and Greece [2], as well as with the findings of Mokhber et al. [18], which reported differences in nutritional risk prevalence between depressed and non-depressed individuals.

Several biological and psychosocial mechanisms may explain these associations. First, vitamin B deficiency may impair neurotransmitter synthesis by elevating homocysteine levels and disrupting one-carbon metabolism [24,25]. Second, insufficient protein intake may lead to tryptophan deficiency, which in turn impairs serotonin synthesis and negatively affects mood regulation. Third, nutritional risk has been associated with elevated levels of inflammatory cytokines, contributing to neuroinflammation [1]. Finally, at the psychosocial level, nutritional risk-related symptoms such as decreased appetite, weight loss, and fatigue may lead to social withdrawal and diminished self-esteem.

The finding that the association between nutritional risk and depression (B = 0.314) was larger in magnitude than that with cognitive function (B = −0.051) may reflect the possibility that depressive symptoms are more sensitive to short-term nutritional and neurochemical changes, whereas cognitive function may represent longer-term cumulative processes. These findings suggest that nutritional status may be more closely related to depressive symptoms; however, direct comparisons of coefficient magnitude should be interpreted with caution.

Nutritional risk showed a weak negative correlation with cognitive function (r = −0.191), and each 1-point increase in the nutritional risk score was associated with a 0.051-point decrease in cognitive function (*p* < 0.01). These findings are consistent with those of Jeong [17] and with Ortega et al.’s [26] report on the association between dietary quality and MMSE scores.

While Jeon [7] reported that nutritional intake among older adults living alone affected cognitive function only indirectly, with depression as a mediator, the present study identified a significant direct association between nutritional risk and cognitive function. This difference may be explained by variations in sample characteristics (older adults living alone vs. all community-dwelling older adults), analytical approaches (mediation analysis vs. regression analysis), and the broader generalizability afforded by a large, nationally representative sample.

The relatively modest association of nutritional risk with cognitive function may reflect the inherent limitations of a cross-sectional study design. Cognitive decline is a long-term process involving progressive brain atrophy and vascular changes; therefore, a single time-point measurement may underestimate this association. Consistent with this interpretation, Kim et al. [24] reported in a longitudinal study that folate and homocysteine levels predicted cognitive decline over time, suggesting that the relationship between nutrition and cognition becomes more evident when a temporal dimension is considered.

With respect to sociodemographic and health-related factors, depression tended to be higher and cognitive function lower among older adults of advanced age, lower educational attainment, living alone, or not currently working. These findings are generally consistent with previous studies [17]. Self-rated health emerged as a strong predictor of both depression and cognitive function, with better perceived health associated with lower depression and higher cognitive function.

Notably, older adults living in urban areas exhibited lower levels of depression, but also lower cognitive function compared with those in rural areas, highlighting the need to identify potentially vulnerable older adults in urban settings. Functional status was also an important factor: older adults who were dependent in activities of daily living (ADL) showed significantly higher depression and lower cognitive function, consistent with the findings of Park et al. [27].

Smoking and alcohol use were not significantly associated with mental health outcomes, which may reflect survivor bias or limitations in measurement. In contrast, disability status was not significantly associated with cognitive function, suggesting that functional dependence may have a more direct relationship with mental health outcomes than disability status per se.

The stronger associations observed among urban residents and non-working older adults suggest potential social and structural vulnerabilities. Urban environments may involve weaker social networks and reduced community cohesion, while lack of economic activity may limit opportunities for social interaction and role engagement. These findings underscore the importance of integrated interventions that address both nutritional and social dimensions of health in older adults.

In subgroup analyses examining whether the associations between nutritional risk and mental health outcomes differed according to sociodemographic and geriatric characteristics, the association of nutritional risk with depression varied across frailty categories, with the strongest association observed in the pre-frail group. This pattern may reflect differences in physiological reserve and disease burden across frailty levels. Pre-frailty may represent a critical window during which vulnerability increases while the potential for recovery remains, supporting the potential cost-effectiveness of early screening and targeted nutritional interventions.

This study contributes to the existing literature by examining the association between nutritional risk and mental health outcomes using a large, nationally representative sample (*N* = 9955). Previous studies have often been limited to specific regions or smaller samples [2,3,28], whereas the present study enhances generalizability and provides more stable estimates by adjusting for a comprehensive set of covariates through hierarchical regression analyses.

Furthermore, the identification of urban residents, pre-frail older adults, and non-working older adults as potentially vulnerable subgroups has important implications for targeted policy interventions. In particular, the stronger association observed in the pre-frail group is consistent with previous evidence on combined nutrition and exercise interventions [29] and highlights the importance of early-stage intervention. These findings support the conceptualization of pre-frailty as a critical window for timely and targeted intervention.

From a policy perspective, these findings underscore the need for integrated approaches to nutritional management in older adults. Given that social isolation and loneliness are recognized as major public health concerns among older populations [30], the observed association between nutritional risk and mental health outcomes suggests the importance of policies that jointly address nutritional, psychological, and social dimensions of health.

In addition, the pronounced vulnerability of the pre-frail group provides practical insight into the timing and targeting of preventive strategies. Building on prior evidence [29], this study supports the integration of frailty assessment (e.g., FRAIL scale) and nutritional screening (e.g., DETERMINE checklist) into routine health checkups for older adults. Such an approach may facilitate the early identification of individuals at combined risk and enable more targeted and efficient allocation of intervention resources.

The observed vulnerability of urban residents suggests the need to reconsider existing support strategies, which have traditionally focused on rural populations. In particular, these findings highlight the importance of strengthening community-based nutritional support and outreach systems to identify and support potentially underserved older adults in urban settings. The interrelated nature of nutritional risk, depression, and cognitive decline further underscores the limitations of fragmented service delivery and supports the need for more integrated approaches that address nutritional, psychological, and cognitive health simultaneously.

In addition, the vulnerability of non-working older adults suggests that economic activity may provide protective benefits through structured daily routines, social interaction, and a sense of role identity. In line with concerns regarding social isolation among older adults [30], these findings support the development of integrated community-based programs that combine nutritional support with opportunities for social participation, particularly for those not engaged in economic activity.

Mantzorou et al. [2] suggested that nutritional assessment could serve as an important tool for mental health screening in older adults. The present study extends this line of research by demonstrating, in a Korean older adult population, that the DETERMINE checklist may serve as an indicator of risk for depression and cognitive decline. These findings support the potential value of integrating mental health assessment with routine nutritional screening in clinical and community settings.

The increased vulnerability observed at the pre-frail stage further highlights the importance of early identification and preventive intervention. Linking frailty screening (e.g., FRAIL scale) with nutritional assessment (e.g., DETERMINE checklist) may help identify individuals at combined risk and facilitate more targeted intervention strategies. In addition, the findings suggest the potential value of community-based care models that connect healthcare services with social support resources, particularly for individuals with multiple vulnerability factors such as non-working status, living alone, and lower educational attainment.

Despite these strengths, several limitations should be acknowledged. First, given the cross-sectional design, causal relationships cannot be established, and the possibility of reverse causation or unmeasured confounding cannot be ruled out. Although multiple covariates were adjusted for, some residual confounding related to socioeconomic status, healthcare access, or environmental factors may remain. Future research utilizing longitudinal data would be valuable for clarifying temporal ordering and better understanding the dynamic relationships among nutritional risk, depression, and cognitive function.

Furthermore, mediation and bidirectional pathways were not formally examined in the present study; consequently, the direct and indirect associations among these variables could not be quantified. Future studies employing structural equation modeling (SEM) or longitudinal or panel data analyses are warranted to explore these mechanisms more comprehensively.

Second, while the DETERMINE checklist is a useful screening tool, it does not capture specific nutrient intake amounts or biochemical indicators such as albumin, vitamins, and homocysteine. In addition, some items in the DETERMINE checklist may conceptually overlap with covariates such as age, functional status, and frailty, which may introduce potential redundancy in the model. Future studies incorporating more detailed nutritional assessments and biomarker data are therefore needed.

Third, the use of self-reported measures introduces the possibility of social desirability and recall bias. Although this was partially mitigated by excluding proxy responses and incorporating objective cognitive assessments (K-MMSE-2), caution is warranted in interpreting these findings.

Finally, although sampling weights were applied, the absence of stratification and primary sampling unit (PSU) identifiers in the dataset limited the ability to fully account for the complex survey design. As a result, standard errors may have been underestimated, and the findings should be interpreted with caution. In addition, some variables were dichotomized for analytical simplicity, which may have led to a loss of information. Therefore, the findings should be interpreted with caution, as alternative explanations related to measurement limitations and unmeasured confounding cannot be fully excluded.

Future research is needed to further clarify the relationship between nutritional management and mental health outcomes in older adults. Longitudinal analyses using multiyear data from the National Survey of Older Koreans would be valuable for identifying the lagged association of nutritional improvement, while studies employing fixed-effects models may help control for time-invariant confounding.

In addition, randomized controlled trials (RCTs) of integrated nutrition and mental health interventions targeting vulnerable groups, such as urban pre-frail older adults, are needed to evaluate effect sizes and cost-effectiveness and to strengthen the evidence base for policy development.

Although potential nonlinear effects of age and educational attainment were considered, the present study focused on parsimonious models for interpretability. Future studies incorporating polynomial or spline-based approaches may further elucidate potential nonlinear relationships.

The exclusion of proxy respondents may also have introduced selection bias, as proxy interviews are more likely to involve individuals with poorer cognitive function or more severe health conditions. As a result, the observed associations—particularly for cognitive outcomes—may underestimate the true magnitude of the relationship.

Furthermore, international comparative research is needed to distinguish culturally and institutionally specific factors from those that are more broadly generalizable, thereby informing the development of context-appropriate intervention models.

Finally, given that multiple comparisons were conducted across subgroup and item-level analyses, some statistically significant findings may reflect chance associations. Therefore, these results should be interpreted with caution and confirmed in future studies.

## 5. Conclusions

Using a nationally representative sample of 9955 older adults, this study indicated that nutritional risk was significantly associated with depression (B = 0.314, *p* < 0.001) and cognitive function (B = −0.051, *p* < 0.001). Significant interaction associations were observed for residential area (cognitive function) and employment status (both outcomes), suggesting heterogeneity in these associations across specific subgroups.

These findings underscore the importance of integrating nutritional management with mental health strategies for older adults. Early screening and targeted support—particularly among socioeconomically and functionally vulnerable groups—may contribute to more comprehensive and effective care. The development of coordinated approaches that address nutrition, depression, and cognitive health within community settings represents a promising direction for future policy and practice.

Taken together, this study contributes to the existing literature by providing nationally representative evidence on the associations between nutritional risk and mental health outcomes in older adults. Future longitudinal studies and intervention-based research will be essential for clarifying causal pathways and informing integrated health policies for this population.

## Figures and Tables

**Table 1:** General characteristics of study participants (*N* = 9955).

Variables	N/M	%/SD
Nutritional risk scale	2.27	2.55
Cognitive Function scale	24.61	4.90
Depression scale	3.12	3.24
Sex		
Male	4384 (44.0)	44.00
Female	5571 (56.0)	56.00
Age		
65~69 years	3465 (34.8)	34.88
70~79 years	4090 (41.1)	41.11
80~89 years	2161 (21.7)	21.77
≥90 years	239 (2.4)	2.44
Residence		
Urban area	7359 (73.9)	73.99
Rural area	2596 (26.1)	26.11
Education level		
≤Primary education	3994 (40.1)	40.11
≤Secondary education	5259 (52.8)	52.88
≥Higher education	701 (7.0)	7.00
Smoking status		
Smoker	940 (9.4)	9.44
Non-smoker	9015 (90.6)	90.66
Drinking status		
Drinker	3745 (37.6)	37.66
Non-drinker	6210 (62.4)	62.44
Self-rated health		
Very good	268 (2.7)	2.77
Good	4036 (40.0)	40.00
Fair	3450 (34.2)	34.22
Poor	1939 (19.2)	19.22
Very poor	263 (2.6)	2.66
Chronic diseases		
0	1401 (14.1)	14.11
1	2211 (22.2)	22.22
2	2798 (28.1)	28.11
≥3	3546 (35.6)	35.66
Employment status		
Employed	3926 (39.4)	39.44
Unemployed	6029 (60.6)	60.66
Household type		
Living alone	3281 (33.0)	33.00
Living with others	6674 (67.0)	67.00
Frailty level		
Robust	6135 (61.6)	61.66
Pre-frail	3127 (31.4)	31.44
Frail	694 (7.0)	7.00
Physical activity		
Yes	5281 (53.0)	53.00
No	4675 (47.0)	47.00
Disability status		
Yes	374 (3.8)	3.88
No	9581 (96.2)	96.22
Medical visits per month		
<2	6580 (66.1)	66.11
≥2	3375 (33.9)	33.99
ADL		
Independent	9172 (92.1)	92.11
Dependent	783 (7.9)	7.99

**Table 2:** Correlation analysis of depression, cognitive function, and nutritional risk.

	Depression	Cognitive Function	Nutritional Risk
Depression	1		
Cognitive	−0.259 **	1	
Function			
Nutritional risk	0.440 **	−0.191 **	1

**: *p* < 0.05.

**Table 3:** Hierarchical regression analysis for depression.

Depression	Model 1	Model 2	Model 3						
Variables	B	B	B	*β*	*95% CI (Lower, Upper)*	*SE*	*p*	*Tolerance*	*VIF*
Sex									
Male	1	1	1						
Female	0.125	−0.158	−0.143	−0.022	(−0.147, −0.139)	0.002	<0.001	0.692	1.445
Age									
65~69 years	1	1	1						
70~79 years	0.509	−0.092	−0.079	−0.012	(−0.083, −0.075)	0.002	<0.001	0.666	1.501
80~89 years	1.277	−0.158	−0.141	−0.018	(−0.146, −0.135)	0.003	<0.001	0.541	1.848
≥90 years	2.508	−0.044	0.080	0.004	(0.069, 0.092)	0.006	<0.001	0.835	1.198
Residence									
Urban area	1	1	1						
Rural area	−0.412	−0.446	−0.376	−0.051	(−0.380, −0.372)	0.002	<0.001	0.895	1.117
Education Level									
≥Higher education	1	1	1						
≤Secondary education	0.634	0.259	0.250	0.039	(0.240, 0.257)	0.003	<0.001	0.243	4.115
≤Primary education	1.255	0.144	0.054	0.008	(0.047, 0.061)	0.004	<0.001	0.209	4.783
Smoking status									
Non-smoker		1	1						
Smoker		0.172	0.063	0.006	(0.057, 0.069)	0.003	<0.001	0.855	1.170
Drinking status									
Non-drinker		1	1						
Drinker		0.095	−0.072	−0.011	(−0.076, −0.068)	0.002	<0.001	0.745	1.343
Self-rated health		−1.078	−0.990	−0.268	(−0.992, −0.987)	0.001	<0.001	0.647	1.546
Chronic Diseases									
0		1	1						
1		0.157	0.096	0.012	(0.090, 0.101)	0.003	<0.001	0.485	2.063
2		0.261	0.096	0.013	(0.090, 0.101)	0.003	<0.001	0.423	2.364
≥3		0.670	0.183	0.027	(0.177, 0.189)	0.003	<0.001	0.328	3.049
Employment Status									
Employed		1	1						
Unemployed		0.548	0.588	0.089	(0.585, 0.592)	0.002	<0.001	0.782	1.279
Household type									
Living with others		1	1						
Living alone		0.324	0.130	0.019	(0.126, 0.134)	0.002	<0.001	0.884	1.131
Physical Activity									
Yes		1	1						
No		0.555	0.533	0.082	(0.530, 0.536)	0.002	<0.001	0.922	1.085
Disability status									
Yes		0.531	0.525	0.031	(0.516, 0.534)	0.005	<0.001	0.928	1.077
No		1	1						
Frailty Level									
Robust		1	1						
Pre-frail		0.842	0.672	0.096	(0.668, 0.676)	0.002	<0.001	0.795	1.259
Frail		2.733	2.227	0.175	(2.219, 2.235)	0.004	<0.001	0.680	1.471
Medical Visits per Month									
<2		1	1						
≥2		0.050	−0.007	−0.001	(−0.011, −0.004)	0.002	<0.001	0.850	1.177
ADL									
Independent		1	1						
Dependent		1.232	0.705	0.059	(0.699, 0.712)	0.004	<0.001	0.767	1.304
Nutritional risk Scale			0.314	0.247	(0.313, 0.315)	0.000	<0.001	0.719	1.391
Adjusted R^2^	0.061	0.330	0.374						
ΔR^2^	0.061	0.270	0.044						

Notes: B = unstandardized regression coefficient; SE = standard error; CI = confidence interval. Model 1 = sociodemographic variables; Model 2 = health-related variables; Model 3 = nutritional factor (nutritional risk).

**Table 4:** Hierarchical regression analysis for cognitive function.

Cognitive Function	Model 1	Model 2	Model 3						
Variables	B	B	B	*β*	*95% CI (Lower, Upper)*	*SE*	*p*	*Tolerance*	*VIF*
Sex									
Male	1	1	1						
Female	−0.678	−0.572	−0.575	−0.058	(−0.581, −0.568)	0.003	<0.001	0.692	1.445
Age									
65~69 years	1	1	1						
70~79 years	−0.825	−0.468	−0.470	−0.047	(−0.477, −0.463)	0.003	<0.001	0.666	1.501
80~89 years	−2.924	−1.969	−1.972	−0.166	(−1.981, −1.963)	0.005	<0.001	0.541	1.848
≥90 years	−5.475	−3.687	−3.707	−0.116	(−3.727, −3.688)	0.010	<0.001	0.835	1.198
Residence									
Urban area	1	1	1						
Rural area	−1.496	−1.531	−1.543	−0.138	(−1.549, −1.536)	0.003	<0.001	0.895	1.117
Education Level									
≥Higher education	1	1	1						
≤Secondary education	−1.508	−1.254	−1.252	−0.128	(−1.263, −1.241)	0.006	<0.001	0.243	4.115
≤Primary education	−3.254	−2.583	−2.568	−0.257	(−2.580, −2.556)	0.006	<0.001	0.209	4.783
Smoking status									
Non-smoker		1	1						
Smoker		−0.281	−0.263	−0.016	(−0.273, −0.253)	0.005	<0.001	0.855	1.170
Drinking status									
Non-drinker		1	1						
Drinker		−0.238	−0.210	−0.021	(−0.217, −0.204)	0.003	<0.001	0.745	1.343
Self-rated health		0.585	0.571	0.102	(0.567, 0.575)	0.002	<0.001	0.647	1.546
Chronic Diseases									
0		1	1						
1		−0.151	−0.141	−0.012	(−0.151, −0.132)	0.005	<0.001	0.485	2.063
2		−0.599	−0.572	−0.053	(−0.582, −0.563)	0.005	<0.001	0.423	2.364
≥3		−0.183	−0.104	−0.010	(−0.114, −0.094)	0.005	<0.001	0.328	3.049
Employment Status									
Employed		1	1						
Unemployed		−0.500	−0.506	−0.050	(−0.512, −0.500)	0.003	<0.001	0.782	1.279
Household type									
Living with others		1	1						
Living alone		−0.406	−0.375	−0.036	(−0.381, −0.368)	0.003	<0.001	0.884	1.131
Physical Activity									
Yes		1	1						
No		−0.455	−0.451	−0.046	(−0.457, −0.445)	0.003	<0.001	0.922	1.085
Disability status									
Yes		0.006	0.007	0.000	(−0.007, 0.022)	0.008	0.329	0.928	1.077
No		1	1						
Frailty Level									
Robust		1	1						
Pre-frail		−0.705	−0.678	−0.064	(−0.684, −0.671)	0.003	<0.001	0.795	1.259
Frail		−1.402	−1.320	−0.069	(−1.333, −1.307)	0.007	<0.001	0.680	1.471
Medical Visits per Month									
<2		1	1						
≥2		0.153	0.162	0.016	(0.156, 0.169)	0.003	<0.001	0.850	1.177
ADL									
Independent		1	1						
Dependent		−1.658	−1.573	−0.086	(−1.584, −1.561)	0.006	<0.001	0.767	1.304
Nutritional risk Scale			−0.051	−0.026	(−0.052, −0.050)	0.001	<0.001	0.719	1.391
Adjusted R^2^	0.204	0.253	0.254						
ΔR^2^	0.204	0.049	0.001						

Notes: B = unstandardized regression coefficient; SE = standard error; CI = confidence interval. Model 1 = sociodemographic variables; Model 2 = health-related variables; Model 3 = nutritional factor (nutritional risk).

**Table 5:** Individual nutritional management items associated with mental health.

Category	Depression			Cognitive Function		
	B	*SE*	*p*	B	*SE*	*p*
Diet modification due to health condition						
Yes	1		1	1		1
No	−0.325	0.081	<0.001	−0.108	0.121	0.370
Less than two meals per day						
Yes	1		1	1		1
No	−1.287	0.205	<0.001	−0.312	0.265	0.240
Rarely consumes fruits, vegetables, or dairy products						
Yes	1		1	1		1
No	−0.557	0.073	<0.001	0.347	0.114	0.002
Drinks three or more alcoholic beverages almost daily						
Yes	1		1	1		1
No	−0.321	0.220	0.144	−0.335	0.242	0.166
Difficulty eating due to poor oral health						
Yes	1		1	1		1
No	−0.403	0.143	0.005	0.181	0.214	0.398
Difficulty purchasing food due to financial constraints						
Yes	1		1	1		1
No	−1.682	0.215	<0.001	0.729	0.283	0.010
Eats alone most of the time						
Yes	1		1	1		1
No	−0.183	0.104	0.078	−0.078	0.163	0.634
Takes three or more different medications daily						
Yes	1		1	1		1
No	−0.081	0.116	0.487	−0.173	0.164	0.292
Unintentional weight change (≥5 kg) in the past six months						
Yes	1		1	1		1
No	−0.598	0.218	0.006	−1.523	0.283	<0.001
Difficulty shopping, cooking, or managing meals						
Yes	1		1	1		1
No	−1.044	0.142	<0.001	0.553	0.211	0.009

Notes: B = unstandardized regression coefficient; SE = standard error. R^2^ = 0.379 (Depression), 0.259 (Cognitive Function).

**Table 6:** Nutritional risk and mental health across sociodemographic and geriatric characteristics by residential area, educational attainment, employment status, frailty status, exercise habits, and ADL.

Moderator	Depression			Cognitive Function		
	B	*SE*	*p*	B	*SE*	*p*
Residence						
Urban area (ref.)						
Rural area	−0.017	0.029	0.571	0.234	0.060	<0.001
Education Level						
≤Primary education	−0.032	0.060	0.595	−0.083	0.061	0.175
≤Secondary education	−0.060	0.059	0.310	0.123	0.058	0.036
≥Higher education (ref.)						
Employment Status						
Employed (ref.)						
Unemployed	0.078	0.030	0.010	−0.106	0.044	0.015
Frailty Level						
Robust (ref.)						
Pre-frail	−0.027	0.032	0.403	−0.072	0.046	0.118
Frail	−0.095	0.052	0.065	0.093	0.069	0.181
Physical Activity						
Yes (ref.)						
No	0.033	0.028	0.230	−0.016	0.041	0.700
ADL						
Independent (ref.)						
Dependent	0.015	0.046	0.749	0.057	0.073	0.432

Notes: B = unstandardized regression coefficient; SE = standard error. Statistical significance was set at *p* < 0.05. Subgroup analyses were exploratory, and *p*-values were not adjusted for multiple comparisons.

## Data Availability

The data used in this study is not publicly available due to privacy and ethical restrictions imposed by the data provider.

## Supplementary Materials

The following supporting information can be downloaded at: https://www.mdpi.com/article/10.3390/healthcare14081062/s1, Supplementary S1: DETERMINE checklist items; Supplementary S2: Geriatric Depression Scale (GDS); Table S1: Differences in depression and cognitive function according to general characteristics and nutritional management items; Table S2: Internal consistency of DETERMINE; Table S3: Internal consistency of GDS-15; Table S4: Scoring Weights of the DETERMINE Checklist; Table S5: Variable coding scheme used in regression analyses. Table S6: Sensitivity analysis of hierarchical regression models for depression and cognitive function; Table S7: Descriptive Statistics, Skewness, and Kurtosis of GDS-15 and K-MMSE-2 Scores.

## Abbreviations

The following abbreviations are used in this manuscript:

ADL	Activities of Daily Living
TAPI	Tablet-PC-Assisted Personal Interview
K-MMSE-2	Korean version of the Mini-Mental State Examination-2
NSI	Nutrition Screening Initiative
