# Supplementary material for: Association Between Nutritional Risk and Mental Health in Older Adults: Focusing on Depression and Cognitive Function
Source: Healthcare (Basel). 2026 Apr 16;14(8):1062. doi: 10.3390/healthcare14081062 (PMC13116351; doi:10.3390/healthcare14081062)
Supplement: Supplementary file 1 [file healthcare-14-01062-s001.zip › healthcare-4208507-supplementary.pdf]

Supplementary S1. DETERMINE checklist items

1. I have an illness or condition that made me change the kind and/or amount of food I eat.
2. I eat fewer than two meals per day.
3. I eat few fruits or vegetables, or milk products.
4. I have 3 or more drinks of beer, liquor, or wine almost every day.
5. I have tooth or mouth problems that make it hard for me to eat.
6. I don’t always have enough money to buy the food I need.
7. I eat alone most of the time.
8. I take 3 or more different prescribed or over-the-counter drugs a day.
9. Without wanting to, I have lost or gained 10 pounds in the last 6 months.
10. I am not always physically able to shop, cook and/or feed myself.

This supplementary material presents the DETERMINE checklist items used to assess nutritional status among older adults.

Supplementary S2. Geriatric Depression Scale(GDS)

1. Are you generally satisfied with your current life?
2. Have you lost a lot of activity or motivation lately?
3. Do you feel like you are living in vain?
4. Does life often feel boring?
5. Do you usually feel refreshed?
6. Are you anxious that something bad will happen to you?
7. Are you generally happy?
8. Do you often feel hopeless?
9. Do you prefer to stay at home rather than going out?
10. Do you feel like your memory is worse than other people your age?
11. Do you find it enjoyable to be alive now?
12. Do you feel like you are worthless right now?
13. Do you have good energy?
14. Do you feel like there is no hope in your situation right now?
15. Do you think you are worse off than other people?

This supplementary material presents the items of the 15-item Geriatric Depression Scale (GDS-15) used to assess depressive symptoms among older adults.

Supplementary Table S1. Differences in depression and cognitive function according to general characteristics and nutritional management items

		Dependent Variable							
		Depression				Cognitive Function			
		M±SD	t / F	p-value	Scheffe	M±SD	t / F	p-value	Scheffe
Sex	Male	2.87±3.13	-6.996***	<0.001		25.49±4.42	16.352***	<0.001	
	Female	3.32±3.31				23.92±5.14			
Age	65~69 (a)	2.40±2.82	164.752***	<0.001	d > c > b > a	26.28±4.31	492.012***	<0.001	a > b > c > d
	70~79 (b)	3.10±3.20				24.88±4.46			
	80~89 (c)	4.07±3.51				22.04±5.04			
	≥90 (d)	5.34±3.81				19.32±5.61			
Region of Residence	Urban area	3.16±3.28	2.335**	0.020		25.18±4.19	16.282***	<0.001	
	Rural area	3.00±3.11				23.03±6.24			
Education Level	≤ Primary education (a)	3.81±3.51	167.781***	<0.001	a > b > c	22.52±4.80	740.110***	<0.001	c > b > a
	≤ Secondary education (b)	2.74±2.97				25.82±4.62			
	≥ Higher education (c)	2.09±2.80				27.50±2.36			
Smoking status in the past year	Smoker	2.92±3.06	-2.096**	0.036		25.41±4.91	5.177***	<0.001	
	Non-smoker	3.14±3.25				24.53±4.89			
Drinking status in the past year	Drinker	2.66±2.96	-11.377***	<0.001		25.45±4.72	13.481***	<0.001	
	Non-drinker	3.40±3.36				24.11±4.94			
Self-rated Health	Very good (a)	1.48±2.05	774. 286***	<0.001	e > d > c > b, a	26.87±2.61	265.718***	<0.001	a > b > c > d > e

	Good (b)	1.76±2.24					26.01±4.12				
	Fair (c)	3.15±2.89					24.25±5.08				
	Poor (d)	5.49±3.61					22.71±4.96				
	Very poor (e)	7.78±3.99					19.70±5.81				
Number of Chronic Diseases	0 (a)	1.67±2.42	348.640	***	<0.001	d > c > b > a	26.18±4.08	121.541	***	<0.001	a > b > c > d
	1 (b)	2.35±2.78					25.40±4.57				
	2 (c)	2.88±2.94					24.49±5.34				
	≥3 (d)	4.37±3.56					23.61±4.79				
Current Employment Status	Employed	2.24±2.58	-23.741	***	<0.001		25.56±4.53	16.025	***	<0.001	
	Unemployed	3.69±3.49					24.00±5.03				
Number of Medical Visits in the Past Month	<2	2.72±3.04	-16.884	***	<0.001		25.01±4.93	11.463	***	<0.001	
	≥2	3.91±3.46					23.85±4.74				
ADL	Independent	2.83±3.00	-25.388	***	<0.001		24.95±4.67	20.486	***	<0.001	
	Dependent	6.47±3.91					20.67±5.69				
Living Arrangement	Living alone	3.76±3.52	13.201	***	<0.001		23.55±5.12	-14.948	***	<0.001	
	Living with others	2.81±3.04					25.14±4.70				
Frailty Level	Robust (a)	2.21±2.55	1181.532	***	<0.001	c > b > a	25.66±4.53	482.242	***	<0.001	a > b > c
	Pre-frail (b)	3.95±3.31					23.40±4.78				
	Frail (c)	7.42±3.85					20.86±5.41				
Regular Physical Activity	Yes	2.62±2.98	-16.325	***	<0.001		25.27±2.98	14.060	***	<0.001	
	No	3.68±3.42					23.88±5.24				

Disability Status	Yes	5.64±4.21	11.887	***	<0.001	22.86±5.82	-5.973	***	<0.001
	No	3.02±3.15				24.68±4.85			
Diet modification due to health condition	Yes	3.82±3.38	-10.862	***	<0.001	24.70±4.45	-0.957		0.338
	No	2.93±3.17				24.59±5.01			
Less than two meals per day	Yes	6.11±4.13	-13.630	***	<0.001	24.02±4.95	2.284	**	0.022
	No	3.02±3.15				24.64±4.90			
Rarely consumes fruits, vegetables, or dairy products	Yes	3.99±3.57	-16.265	***	<0.001	23.97±4.90	8.433	***	<0.001
	No	2.76±3.02				24.88±4.87			
Drinks three or more alcoholic beverages almost daily	Yes	3.62±3.56	-2.568	**	0.011	25.23±4.00	-2.737	**	0.007
	No	3.10±4.92				24.60±4.92			
Difficulty eating due to poor oral health	Yes	5.81±3.83	-19.858	***	<0.001	22.04±5.34	13.552	***	<0.001
	No	2.90±3.09				24.81±4.80			
Difficulty purchasing food due to financial constraints	Yes	7.26±3.88	-21.343	***	<0.001	21.87±4.82	11.311	***	<0.001
	No	2.96±3.10				24.73±4.87			
Eats alone most of the time	Yes	4.28±3.78	-16.559	***	<0.001	23.22±4.91	14.831	***	<0.001
	No	2.81±3.00				24.99±4.83			
Takes three or more different medications daily	Yes	4.41±3.64	-26.102	***	<0.001	23.60±4.80	14.523	***	<0.001
	No	2.51±2.83				25.10±4.87			
Unintentional weight change (≥5 kg) in the past six months	Yes	5.31±3.84	-9.465	***	<0.001	25.15±4.66	-1.786	*	0.074
	No	3.06±3.20				24.60±4.90			
Difficulty shopping, cooking,	Yes	6.26±3.93	-28.598	***	<0.001	21.74±5.44	18.710	***	<0.001

or managing meals	No	2.74±2.92	24.97±4.71

Supplementary Table S1. Differences in Mental Health According to General Characteristics and Nutritional Management Items

The results of the analysis of the differences in depression and cognitive function according to general characteristics and nutritional management items are presented in Table 2.

S1.1. Differences According to General Characteristics

With respect to age, those aged 90 years and older had depression scores of 5.34 (SD = 3.81) and cognitive function scores of 19.32 (SD = 5.61), while those aged 80–89 years had scores of 4.07 (SD = 3.51) and 22.04 (SD = 5.04), respectively. Those aged 65–69 years had the lowest depression scores of 2.40 (SD = 2.82) and the highest cognitive function scores of 26.28 (SD = 4.31). Post-hoc analysis revealed that depression was highest among those aged 90 years and older, followed by those in their 80s, 70s, and 60s in descending order. Cognitive function showed the opposite pattern, being highest in the 60s age group and lowest in the 90s age group.

Educational attainment exhibited a similar pattern. Those with primary education had depression scores of 3.81 (SD = 3.51) and cognitive function scores of 22.52 (SD = 4.80), while those with tertiary education had scores of 2.09 (SD = 2.80) and 27.50 (SD = 2.36), respectively. Post-hoc analysis indicated that depression was highest among those with primary education and lowest among those with tertiary education, whereas cognitive function showed the opposite pattern.

Regarding perceived health status, those reporting "very good" health had depression scores of 1.48 (SD = 2.05) and cognitive function scores of 26.87 (SD = 2.61), those reporting "good" health had scores of 1.76 (SD = 2.24) and 26.01 (SD = 4.12), and those reporting "very poor" health had scores of 7.78 (SD = 3.99) and 19.70 (SD = 5.81), respectively. Post-hoc analysis revealed that depression was highest among those reporting "very poor" health, with no significant difference between "very good" and "good" groups. For cognitive function, scores were highest among those reporting "very good" health, followed by "good," with "very poor" showing the lowest scores.

With respect to chronic diseases, those with no chronic diseases had depression scores of 1.67 (SD = 2.42) and cognitive function scores of 26.18 (SD = 4.08), while those with 3 or more chronic diseases had scores of 4.37 (SD = 3.56) and 23.61 (SD = 4.79), respectively. Post-hoc analysis indicated that depression was highest among those with three or more chronic diseases, while cognitive function was highest among those with no chronic diseases.

Regarding frailty status, robust older adults had depression scores of 2.21 (SD = 2.55) and cognitive function scores of 25.66 (SD = 4.53), pre-frail older adults had scores of 3.95 (SD = 3.31) and 23.40 (SD = 4.78), and frail older adults had scores of 7.42 (SD = 3.85) and 20.86 (SD = 5.41), respectively. Post-hoc analysis revealed that depression was highest among frail older adults, followed by the pre-frail and robust groups. Cognitive function showed the reverse order: robust, pre-frail, and frail.

S1.2. Differences According to Nutritional Management Items

Analysis of differences in nutritional management items revealed that depression scores were generally higher among those who responded "yes" to each item. However, for the item "drinking 3 or more glasses of alcohol almost every day," the "no" response group showed higher depression scores. Similarly, for cognitive function, "yes" responses were associated with lower scores for most items, but the "drinking 3 or more glasses of alcohol almost every day" item showed significantly higher cognitive function scores in the "no" response group. Although a few items such as "changing the amount or type of food" were not statistically significant for cognitive function, most items showed significant results.

Supplementary Table S2. Internal consistency of DETERMINE

DETERMINE<br>Item	Corrected item–total correlation	α if item deleted
1. I have an illness or condition that made me change the kind and/or amount of food I eat.	0.162	0.454
2. I eat fewer than two meals per day.	0.110	0.470
3. I eat few fruits or vegetables, or milk products.	0.228	0.434
4. I have 3 or more drinks of beer, liquor, or wine almost every day.	0.056	0.466
5. I have tooth or mouth problems that make it hard for me to eat.	0.278	0.411
6. I don't always have enough money to buy the food I need.	0.305	0.417
7. I eat alone most of the time.	0.207	0.429
8. I take 3 or more different prescribed or over-the-counter drugs a day.	0.223	0.425
9. Without wanting to, I have lost or gained 10 pounds in the last 6 months.	0.146	0.451
10. I am not always physically able to shop, cook and/or feed myself.	0.320	0.390

Overall α : 0.462

Supplementary Table S3. Internal consistency of GDS-15

Geriatric Depression Scale(GDS)		
Item	Corrected item–total correlation	α if item deleted
1. Are you generally satisfied with your current life?	0.560	0.825
2. Have you lost a lot of activity or motivation lately?	0.504	0.828
3. Do you feel like you are living in vain?	0.399	0.834
4. Does life often feel boring?	0.506	0.828
5. Do you usually feel refreshed?	0.529	0.826
6. Are you anxious that something bad will happen to you?	0.419	0.833
7. Are you generally happy?	0.525	0.827
8. Do you often feel hopeless?	0.479	0.831
9. Do you prefer to stay at home rather than going out?	0.442	0.832
10. Do you feel like your memory is worse than other people your age?	0.418	0.833
11. Do you find it enjoyable to be alive now?	0.461	0.831
12. Do you feel like you are worthless right now?	0.432	0.833
13. Do you have good energy?	0.398	0.837
14. Do you feel like there is no hope in your situation right now?	0.519	0.829
15. Do you think you are worse off than other people?	0.499	0.829

Overall α : 0.840

Supplementary Table S4. Scoring Weights of the DETERMINE Checklist

DETERMINE		
Item	Response (Yes=1)	Assigned Weight
1. I have an illness or condition that made me change the kind and/or amount of food I eat.	Yes/No	2
2. I eat fewer than two meals per day.	Yes/No	3
3. I eat few fruits or vegetables, or milk products.	Yes/No	2
4. I have 3 or more drinks of beer, liquor, or wine almost every day.	Yes/No	2
5. I have tooth or mouth problems that make it hard for me to eat.	Yes/No	2
6. I don't always have enough money to buy the food I need.	Yes/No	4
7. I eat alone most of the time.	Yes/No	1
8. I take 3 or more different prescribed or over-the-counter drugs a day.	Yes/No	1
9. Without wanting to, I have lost or gained 10 pounds in the last 6 months.	Yes/No	2
10. I am not always physically able to shop, cook and/or feed myself.	Yes/No	2

Supplementary Table S5. Variable coding scheme used in regression analyses

Variable	Category	Coding
Sex	Male	0(reference)
	Female	1
Age	65~69	0(reference)
	70~79	Dummy 1
	80~89	Dummy 2
	≥90	Dummy 3
Region of Residence	Urban area	0(reference)
	Rural area	Dummy
Education Level	≤ Primary education	Dummy 2
	≤ Secondary education	Dummy 1
	≥ Higher education	0(reference)
Smoking status in the past year	Smoker	1
	Non-smoker	0(reference)
Drinking status in the past year	Drinker	1
	Non-drinker	0(reference)
Self-rated Health	Very good	5
	Good	4
	Fair	3
	Poor	2
	Very poor	1
Number of Chronic Diseases	0	0(reference)

	1	Dummy 1
	2	Dummy 2
	≥3	Dummy 3
Current Employment Status	Employed	0(reference)
	Unemployed	1
Number of Medical Visits in the Past Month	<2	0(reference)
	≥2	1
ADL	Independent	0(reference)
	Dependent	1
Living Arrangement	Living alone	1
	Living with others	0(reference)
Frailty Level	Robust	0(reference)
	Pre-frail	Dummy 1
	Frail	Dummy 2
Regular Physical Activity	Yes	0(reference)
	No	1
Disability Status	Yes	0(reference)
	No	1
Diet modification due to health condition	Yes	0(reference)
	No	1
Less than two meals per day	Yes	0(reference)
	No	1
Rarely consumes fruits, vegetables, or dairy products	Yes	0(reference)
	No	1
Drinks three or more alcoholic beverages almost daily	Yes	0(reference)
	No	1

Difficulty eating<br>due to poor oral health	Yes<br>No	0(reference)<br>1
Difficulty purchasing food<br>due to financial constraints	Yes<br>No	0(reference)<br>1
Eats alone most of the time	Yes<br>No	0(reference)<br>1
Takes three or more different<br>medications daily	Yes<br>No	0(reference)<br>1
Unintentional weight change (≥5 kg)<br>in the past six months	Yes<br>No	0(reference)<br>1
Difficulty shopping, cooking,<br>or managing meals	Yes<br>No	0(reference)<br>1
Nutritional risk scale	Continuous (0–21)	Higher = poorer nutrition
Cognitive Function scale	Continuous (0–15)	Higher = more depressive symptoms
Depression scale	Continuous (0–30)	Higher = better function

Supplementary Table S6. Sensitivity analysis of hierarchical regression models for depression and cognitive function

Variables		Depression			Cognitive Function		
		Main model	Medical Visits per Month	ADL	Main model	Medical Visits per Month	ADL
		B	B	B	B	B	B
Sex	Female	-0.143	-0.140	-0.144	-0.575	-0.570	-0.566
Age	70~79 years	-0.079	-0.088	-0.083	-0.470	-0.476	-0.482
	80~89 years	-0.141	-0.145	-0.126	-1.972	-1.976	-2.011
	≥90 years	0.080	0.057	0.036	-3.707	-3.716	-3.687
Residence	Rural area	-0.376	-0.416	-0.413	-1.543	-1.545	-1.536
Education Level	≤ Secondary education	0.250	0.266	0.268	-1.252	-1.249	-1.258
	≤ Primary education	0.054	0.079	0.083	-2.568	-2.576	-2.585
Smoking status	Smoker	0.063	0.115	0.122	-0.263	-0.289	-0.297
Drinking status	Drinker	-0.072	-0.049	-0.049	-0.210	-0.222	-0.230
	Self-rated health	-0.990	-1.015	-1.002	0.571	0.579	0.549
Chronic Diseases	1	0.096	0.130	0.132	-0.141	-0.182	-0.149
	2	0.096	0.154	0.167	-0.572	-0.633	-0.623
	≥3	0.183	0.290	0.313	-0.104	-0.248	-0.238
Employment Status	Unemployed	0.588	0.567	0.574	-0.506	-0.494	-0.507
Household type	Living alone	0.130	0.172	0.187	-0.375	-0.404	-0.425
Physical Activity	No	0.533	0.550	0.549	-0.451	-0.448	-0.449
Disability status	Yes	0.525	0.507	0.474	0.007	-0.005	0.070
Frailty Level	Pre-frail	0.672	0.689	0.701	-0.678	-0.703	-0.718
	Frail	2.227	2.290	2.239	-1.320	-1.368	-1.233
Nutritional risk	(continuous)	0.314			-0.051		

	Moderate risk		0.762	0.786		0.172	0.130
	High risk		2.271	2.326		-0.273	-0.400
Medical Visits per Month	(continuous)	0.162	0.012	0.016	0.162	0.057	0.125
ADL	(continuous)	-1.573	0.763	-0.259	-1.573	-1.626	0.535

Supplementary Table S7. Descriptive Statistics, Skewness, and Kurtosis of GDS-15 and K-MMSE-2 Scores

Variable	Mean ± SD	Skewness	Kurtosis
GDS-15 (total score)	3.09 ± 3.24	1.26	1.17
K-MMSE-2 (total score)	24.50 ± 4.77	-1.58	3.86
